# MICROBIOME AND AGING: INSIGHTS INTO COGNITIVE HEALTH FROM THE MIAGB CONSORTIUM

**DOI:** 10.1093/geroni/igae098.1594

**Published:** 2024-12-31

**Authors:** Hariom Yadav

**Affiliations:** University of South Florida, Tampa, Florida, United States

## Abstract

World population is aging, and aging-related conditions are on the rise, with no better prognosis, prevention, and treatment. Because we do not fully understand the complex biology of aging and its dysfunctional attributes, it contributes to aging-related conditions. Emerging evidence shows that the gut microbiome plays a critical role in the biology of aging and has thus been proposed to be one of the targets for Geroscience-based interventions. However, we still lack understanding of how the microbiome contributes to aging and its related disorders. To address these knowledge gaps, a collective effort of several institutions has evolved a multi-center clinical study called Microbiome in Aging Gut and Brain (MiaGB), which has focused on developing large datasets on the microbiome and its related pathways, including metabolomics, proteomics, epigenomics, immunophenomics, and others, to understand how the microbiome contributes to aging biology, thus impacting brain health and overall health linked with aging. Dr. Yadav will present the outcomes of the MiaGB consortium addressing how the microbiome contributes to aging-related cognitive decline by impacting intestinal epithelial barriers, inflammation, and immune functions. He will also emphasize whether and how reversing these microbiome-related abnormalities can ameliorate aging-related disorders in older adults.

